# Supplementary figures and images for: Etravirine Prevents West Nile Virus and Chikungunya Virus Infection Both In Vitro and In Vivo by Inhibiting Viral Replication
Source: Pharmaceutics. 2024 Aug 23;16(9):1111. doi: 10.3390/pharmaceutics16091111 (PMC11435157; doi:10.3390/pharmaceutics16091111)

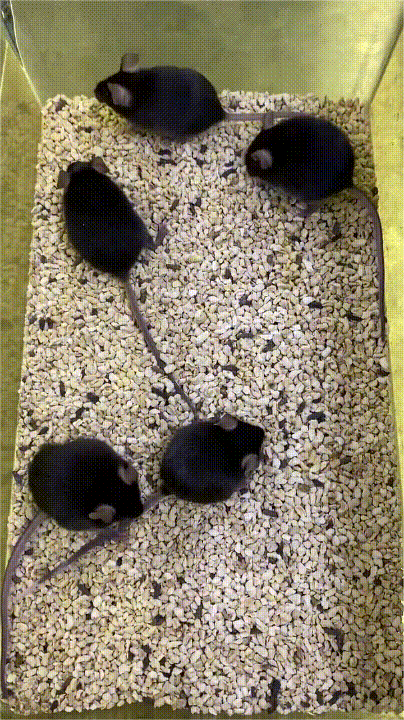

Supplement: Supplementary file 1 [file pharmaceutics-16-01111-s001.zip › Figure S1/DMSO.gif]

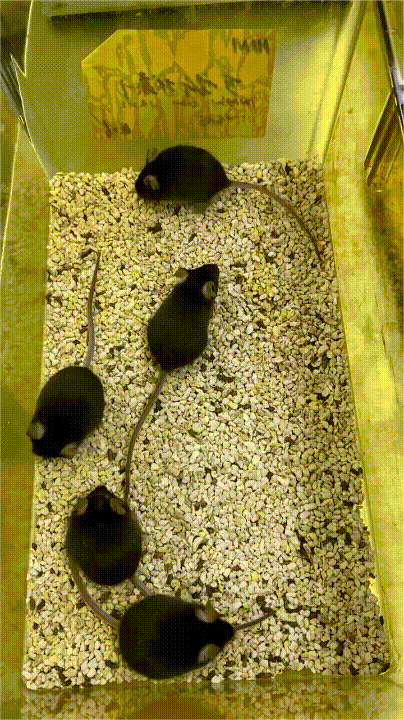

Supplement: Supplementary file 1 [file pharmaceutics-16-01111-s001.zip › Figure S1/ETR.gif]

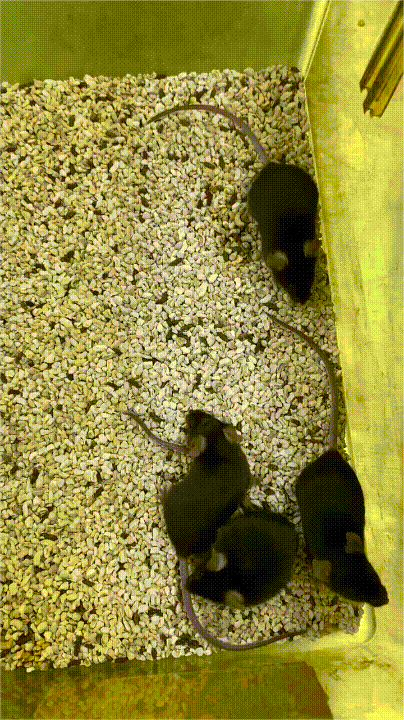

Supplement: Supplementary file 1 [file pharmaceutics-16-01111-s001.zip › Figure S1/mock.gif]

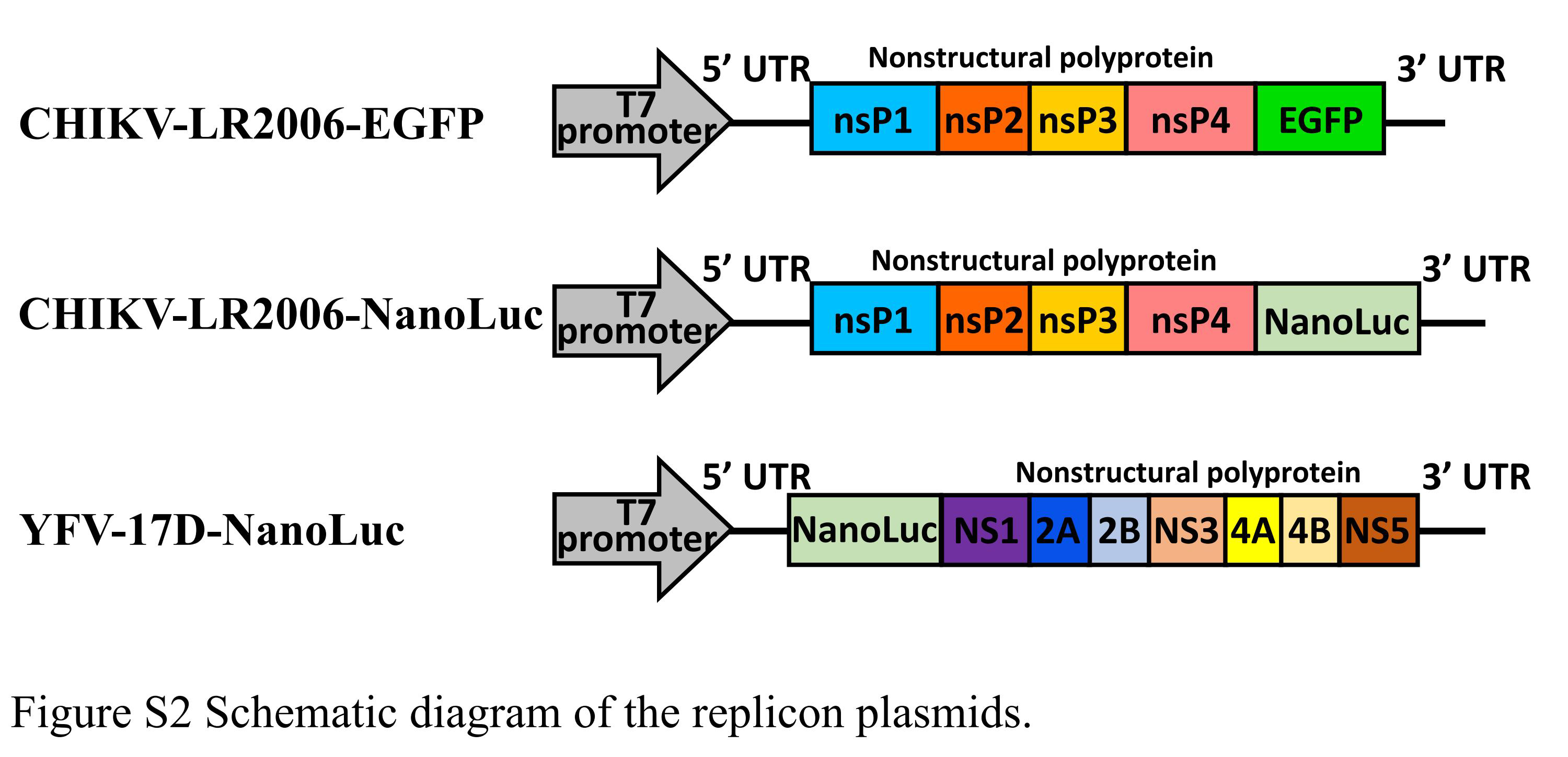

Supplement: Supplementary file 1 [file pharmaceutics-16-01111-s001.zip › Figure S2.jpg]
